# Accuracy of WAAS-Enabled GPS-RF Warning Signals When Crossing a Terrestrial Geofence

**DOI:** 10.3390/s16060912

**Published:** 2016-06-18

**Authors:** Lindsay M. Grayson, Robert F. Keefe, Wade T. Tinkham, Jan U. H. Eitel, Jarred D. Saralecos, Alistair M. S. Smith, Eloise G. Zimbelman

**Affiliations:** 1 Department of Forest, Rangeland and Fire Sciences, University of Idaho, 875 Perimeter Drive, Moscow, ID 83844-1133, USA; gray7728@vandals.uidaho.edu (L.M.G.); jeitel@uidaho.edu (J.U.H.E.); alistair@uidaho.edu (A.M.S.S.); eloisez@uidaho.edu (E.G.Z.); 2 Department of Forest and Rangeland Stewardship, Colorado State University, 1472 Campus Delivery, Fort Collins, CO 80523, USA; wade.tinkham@colostate.edu; 3 Department of Forest Management, University of Montana, 32 Campus Drive, Missoula, MT 59812, USA; jarred.saralecos@umontana.edu

**Keywords:** geofence, virtual fence, real-time GPS, GPS-RF, GNSS, GNSS-RF, position, navigation and timing

## Abstract

Geofences are virtual boundaries based on geographic coordinates. When combined with global position system (GPS), or more generally global navigation satellite system (GNSS) transmitters, geofences provide a powerful tool for monitoring the location and movements of objects of interest through proximity alarms. However, the accuracy of geofence alarms in GNSS-radio frequency (GNSS-RF) transmitter receiver systems has not been tested. To achieve these goals, a cart with a GNSS-RF locator was run on a straight path in a balanced factorial experiment with three levels of cart speed, three angles of geofence intersection, three receiver distances from the track, and three replicates. Locator speed, receiver distance and geofence intersection angle all affected geofence alarm accuracy in an analysis of variance (*p* = 0.013, *p* = 2.58 × 10^−8^, and *p* = 0.0006, respectively), as did all treatment interactions (*p* < 0.0001). Slower locator speed, acute geofence intersection angle, and closest receiver distance were associated with reduced accuracy of geofence alerts.

## 1. Introduction

Geofences are virtual boundaries marked by geographic coordinates, typically set either as a circle of a given radius from a central point or as a polygon whose vertices are predetermined by an operator. Many disciplines now use global navigation satellite system (GNSS) tracking to monitor objects or organisms of interest. Devices equipped and used in North America where Wide Area Augmentation System (WAAS) differential correction is available are considered accurate to within 3 m at least 95 percent of the time [1], although individual receiver accuracy depends heavily on the operating environment in which it is used. Geofences coupled with GNSS tracking have been deployed in remote monitoring of sites for security purposes, tracking patients with Alzheimer’s disease, wildlife encroachment onto farmland, alerting guards to the escape of prisoners, ensuring children stay in safe areas, creating security boundaries for wireless signals, transportation management, and tagging animals covering large ranges in remote locations [2,3,4,5,6]. New uses of geofences in wildlife management are developing [7,8]. An emerging application area for global position system (GPS) tracking is forestry, where multi transmitter GPS systems may be useful for logging safety, boundary and silvicultural marking, controlling herbicide applications, and production and cost tracking [9]. Multiple descriptors for geofences exist in the literature [10,11]. In practice, geofences are linear, circular, or polygonal boundaries defined in a Geographic Information System. GNSS receivers on location devices transmit their coordinates to a handheld receiver using radio frequency (RF) such as very high frequency (30 MHz–300 MHz), ultra high frequency (300 MHz–3 GHz), or other bands. The handheld device then provides both a mapping display in near real-time and processing capability to monitor the location device’s position relative to the geofence boundary. Crossing of the geofence in one direction or multiple directions, triggers a visual, audible, or text alert signal on the handheld device, the location device, or both. Agricultural applications have conventionally used the term *virtual fences* [7,12,13,14,15]. The terminology of virtual fences has also been used in studies seeking to promote safety within construction sites, through GNSS tracking of personnel and heavy equipment [16,17]. Virtual fences may be paired with or serve as a component in map matching, which is the process of relating geospatial features such as roads or buildings with GNSS coordinates collected in real-time or near real-time. The map matching process is an important component of GNSS navigation systems [18,19].

Despite the growing use of GNSS tracking and virtual fences for many applications, studies are limited that have documented the accuracy of geofence alerts for moving objects in a replicated, designed experiment. The objective of this study is to determine the temporal accuracy of a commercially-available, recreation-grade GNSS-radio frequency (GNSS-RF) unit’s geofence alert system. Specific research questions we sought to address include an assessment of how dependent the accuracy is on (i) the speed of the tracked object; (ii) the angle at which the object intersects the geofence; and (iii) the distance of the object from the receiver. It is important to note that the scope of our evaluation is limited to the particular recreational GNSS-RF system used. The range of speeds we chose to evaluate corresponds to possible speeds realized by a rubber-tired log skidder or a log truck entering or leaving an operational timber harvest.

## 2. Methods

**Figure 1:**
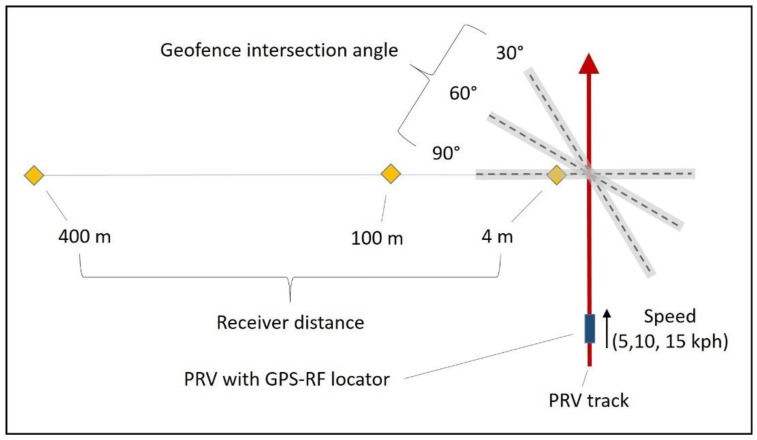
Experimental track, geofences (gray, dashed lines), and receiver locations (gold diamonds). Positions are not to scale. The portable recreational vehicle (PRV) with position tracking traveled from magnetic West to East. All distances and angles were measured using a total station.

In order to quantify sources of variability in geofence crossing signals, a 3 × 3 × 3 factorial experiment was conducted in a clear area under open skies using a motorized portable recreational vehicle (PRV) designed to run on a wooden track at the University of Idaho Forest Operations Research Lab in Princeton, Idaho (46.916096°N–116.831736°W). The experiment was conducted in an open area in order to isolate factors of interest from the potential effects of forest canopy, which are being evaluated in a separate experiment. The recreational vehicle was a motorized cart elevated on a track constructed of pressure-treated structural lumber (3.81 cm × 13.97 cm). The cart was coupled to the track using angle irons with vertical and horizontal wheels, providing a smooth, straight course for evaluation of position, navigation and timing with minimal influence due to variability of the ground surface. The experiment had three levels of vehicle speed (5, 10, and 15 km/h), three levels of geofence intersection angle (30 degrees, 60 degrees, and 90 degrees), three levels of receiver distance perpendicular to the track (4 m, 100 m, and 400 m), and 3 replicates for a total of 81 trials. The track was 60.96 cm in width, and was leveled to less than 10 cm height difference along the 120 m length used for the experiment. It was aligned on the magnetic East/West axis using a total station. The track was flagged at 10 m increments. A WAAS-enabled GNSS-RF transmitter (TT 15, Garmin, United States) was attached via its collar to the front bumper of the PRV, centered on both the bumper and the track approximately 15 cm above the track. The handheld receiver with extended antenna (Alpha 100, Garmin, United States) was attached via a cardboard holder and zip-ties at a height of 1.58 m to a PVC pole driven into the ground at a set 5 m distance perpendicular to the 80 m point along the track, as measured by a total station. The receiver and transmitter were separated only by unobstructed flat land to avoid signal interruptions or reroutes that could affect the transmission times. The geofence was centered on the track at 80 m, and geofence angles were sighted using a total station [20]. For each angle, the end points of the geofence were set to 20 m from the track (Figure 1). Because the Garmin Alpha handheld unit requires polygonal or radius geofences, and not lines, the extraneous points were set 20 m out, 10 m before the start of the track. Points were added to the geofence in the receiver as an average of three waypoints at each location. A calibrated speedometer was mounted to the PRV to provide speed feedback to the driver and was validated through hand timing.

Trials began with an audio signal given when the front bumper of the PRV crossed the first 10 m mark, providing the vehicle 10 m to reach a consistent speed, and ended when the vehicle crossed the 80 m mark completing the 70 m run. A person standing along the transect through the track path manually timed the duration of the 70 m run, with another timing the first 40 m to verify speed over the course of the trial. A third person standing by the receiver timed duration from when the signal was heard to when the geofence crossing alarm sounded on the receiver. Trials were only run in the West to East direction, with the PRV removed from the track and transported via truck back to the starting end for each trial. The time difference between the manual timing at the track and the time recorded with the geofence alarm was computed for each trial as the global dependent variable, with all measurements recorded in seconds. All statistical tests were evaluated at a significance level of α = 0.05 or lower.

## 3. Results

Position locator speed (*p* = 0.013), handheld receiver distance (*p* = 2.58 × 10^−8^) and intersection angle (*p* = 0.0006) affected the time delay between actual geofence crossing and signal reception (Table 1). The interaction of receiver distance and speed (*p* = 2.74 × 10^−5^), receiver distance and intersection angle (*p* = 0.0036), speed and intersection angle (*p* = 0.0025) and of all combinations (*p* = 0.0002) were all significant at the *p* < 0.01 level based on the ANOVA likelihood ratio test (F-test).

**Figure 2:**
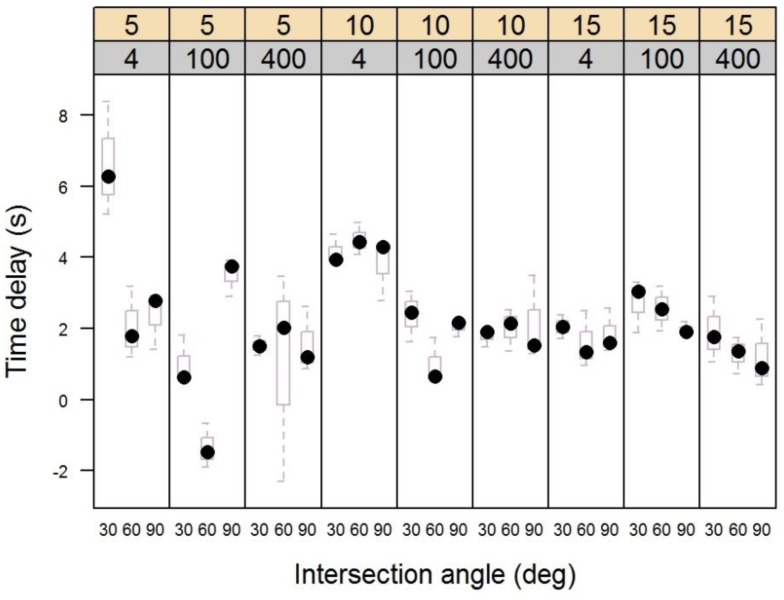
Box-and-whiskers plot showing the time difference between actual run time and the time as measured with the geofence crossing signal. Upper panel numbers are speed (5 kph, 10 kph, 15 kph). Sub-panel scale is receiver distance (4 m, 100 m, 400 m).

Figure 2 shows that across all treatments, the total range of delay times varied inversely with speed, declining for the 5 kph, 10 kph, and 15 kph treatments (10.7 s, 4.47 s, and 2.9 s, respectively). Thus, as the vehicle and position locator moved more quickly, error in the geofence crossing signal was reduced rather than increased. The 30 degree intersection angle had the highest median delay for 5 of the 9 speed and receiver distance treatment combinations, followed by the 60 degree angle for 3 of the remaining. The 90 degree angle median delay was highest for only one treatment combination.

Across all treatments, the widest range of values occurred for the 4m receiver distance. The range in values for the 4 m distance was 7.42 s, followed by 5.81 s and 5.80 s for the 100 m and 400 m distances, respectively. The highest median and interquartile range (IQR) values for any combination of treatment levels occurred at the 4 m receiver distance and 5 kph speed, with a median delay of 6.29 s. The IQR is the range of values that occurs between the 25th and 75th percentiles of the data, and corresponds to the height of the boxes in Figure 2. Across all treatment level combinations, the four highest median delay values occurred at the 4 m receiver distance. However, as with the overall range of delay times observed, the median delay time at the 4 m receiver distance was lowest at the fastest speed observed.

**Figure 3:**
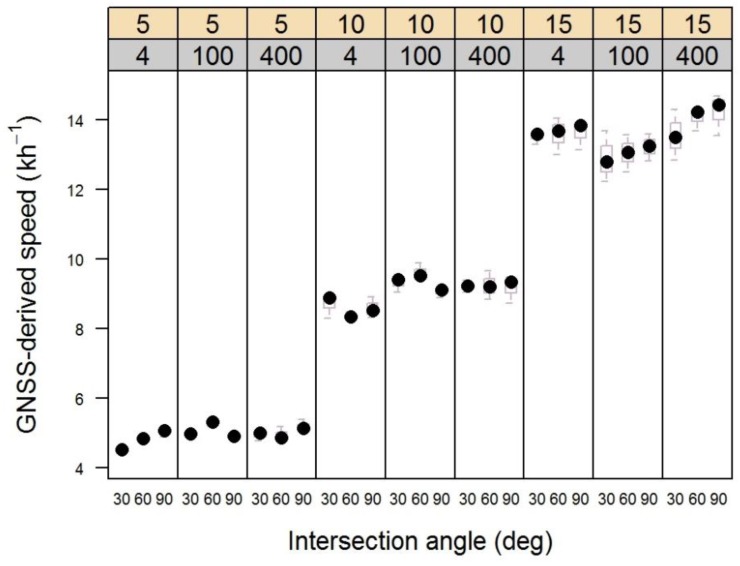
Box-and-whiskers plot showing the distribution of GNSS-based cart speeds for all trials in the experiment. Upper panel numbers are speed (5 kph, 10 kph, 15 kph). Sub-panel scale is receiver distance (4 m, 100 m, 400 m).

Variability in the speed of the cart used in the experiment was very low within each treatment (speed) level, as shown in Figure 3. The highest variability occurred in the fastest (15 kh^−1^). However, there were no obvious trends that might lead to misinterpretation of the time lag delay distributions shown in Figure 2. 

## 4. Discussion

The most notable aspect of our results was the tendency for geofence alerts to have high variability as transmitters crossed the virtual boundary at the slowest speed (5 kph). Most equipment used in operational forestry travels slowly (<10 kph) while working in the woods, e.g., when skidding [21]. This is generally true for feller-bunchers, processors, loaders, skidders and forwarders. All need to travel slowly as they lift or drag heavy loads and navigate over stumps, logs and other irregular surfaces. Similarly, ground workers such as manual fallers and rigging crew staff on cable logging operations travel at walking speed. Basic GIS mapping interfaces are currently becoming available for several major forestry equipment producers, and geofences may be used to delineate harvest unit boundaries or riparian management zones (RMZs). Our results suggest that intersection alerts associated with these virtual boundaries are likely to be insensitive to equipment movements at slow speeds, or to ground workers on foot. Many states and territories have riparian management laws that limit ground-based equipment movements within the SMZ. Until better methods are derived, geofences positioned absolutely at the SMZ boundary should not be relied on for forest practices rule compliance.

The most acute geofence intersection angle, 30°, tended to have a higher time lag between crossing and alert signal than the 60° or 90° intersection angles. This occurred in 55% of all treatment combinations of receiver distance and speed. While it seems plausible that an acute angle might result in early warnings as a transmitter converges gradually toward the virtual boundary, the opposite was true in our experiment. In no case was the alert triggered prior to intersection. One explanation for this is the tendency for the decimal precision of the coordinate reference system as the cart moved west to east on our test track. Coordinates reported by the Garmin Alpha software tend to pixelate along north-south and east-west traverse directions, rather than forming perfectly smoothed lines. At 30°, the reported TT15 position may fall directly in-line with the geofence for a brief period before shifting to the next line of pixels where it appears to have crossed outside.

It is unclear why there appeared to be increased alert time delay at the 4 m receiver distance, as compared with 100 m and 400 m. However, high error rates observed at the closest receiver distance (4 m) have important implications for operational forestry and other natural resource applications involving large, mechanized equipment working in close proximity with ground workers, especially if geofences alerts are used for safety applications. One practical, heuristic solution to account for delayed signal alerts would be to integrate early warning triggers in geofence algorithms. That is, rather than signaling when a crossing has occurred, the algorithm might provide fair warning ahead of time. It may thus be beneficial to conduct a more detailed analysis over a range of relatively short distances in order to better characterize issues associated with position trackers and receivers working in close proximity.

In related studies, the Garmin TT 15 transmitters used in our study have primarily been deployed experimentally on the outside of the equipment cabs. They make it possible for a vibration, text message, or audible signal to signal an alert on the handheld (inside the cab) when a geofence has been crossed. Alpha 100 handheld receivers are small, so visual indicators of location may be difficult for operators to see while working. For future applications in forestry, GNSS positions would presumably be fed directly into onboard computers that are in most conventional agricultural and forestry machinery, in order to provide geofence status alerts to equipment operators [9]. Existing onboard software used by many equipment manufacturers could display GNSS and geofence position and timing for multiple pieces of equipment or ground workers. Or supplemental screens (e.g., tablets) could be used to provide a separate real-time map display with geofence status. Foresters or operators could upload the shapefiles associated with timber sale unit boundaries and use the stand perimeter as a geofence, in order to minimize the potential for contractors to stray outside of units during harvesting. Streamside management zones could be similarly delineated. Safety zones surrounding semi-stationary equipment (e.g., processors) could also be established, especially on cable operations with multiple ground workers [9].

There are a few potential limitations of our study that are worth noting. First, the track and PRV used in our experiment provided highly controlled movements. The PRV traveled more smoothly above the ground surface than a log skidder, feller-buncher, or other piece of heavy equipment used in logging typically would. By design, the experiment was also conducted in an open area that was largely unobstructed by forest canopy or other sources of GNSS multipath or radio propagation error. As use of positioning technology with geofences increases, it will be important to evaluate the effects of ground surface roughness, topography, canopy cover, and other factors on how GNSS-RF positioning interacts with geofence alert signals. Although accuracy assessments of consumer-grade GNSS use in forestry have been studied previously [21,22], GNSS-RF systems have, by necessity, two transmitted signals that may be affected due to GNSS multipath, radio propagation, and other error sources in adverse conditions. More advanced GNSS-RF systems, such as those used for military applications, should also be evaluated for comparison. Systems with higher wattage radio transceivers, higher accuracy GNSS receivers, or higher frequency transmission rates could be less affected by the sources of variability identified in this study. However, an important benefit of low-cost devices as that they are likely to be more accessible to a wide potential user base.

In addition to variability among alternative GNSS-RF systems, it is also important to note that a number of other emerging technological solutions to improve position, navigation and timing, as well as other analytical approaches, may also be useful for applications in forested environments. For example, GNSS-INS systems link GNSS guidance with inertial navigation systems based on accelerometers [23] and other collaborative navigation approaches [24]. Solutions that improve navigation under the canopy by linking GNSS with Light Detection and Ranging (LiDAR) data have been proposed [25], as well as approaches that use multi-resolution digital terrain models [26].

## 5. Conclusions

We have shown that several factors matter when considering the deployment of geofence alert systems in real-time tracking applications. In our experiment, geofence intersection angle, speed, and the distance between receiver and transmitter all affected the accuracy of geofence alarms, as did all interactions of these factors. Development of geofence applications for forestry and other natural resource fields that use consumer-grade positioning systems may need to account for mobile position transmitters with geofences functioning less accurately at moderate speeds, at acute geofence intersection angles, and when receivers are in close proximity. While the error associated with boundary alerts may seem small (e.g., 5 m or less), many applications in logging safety and forest practices monitoring will require more sensitive alert response times or alternative technology in order to be effective. It is important to note that we used a low-cost consumer grade recreational GNSS-RF system for this preliminary research. Subsequent studies should build on this preliminary work to evaluate and compare and contrast other systems.

## Figures and Tables

**Table 1:** ANOVA results for full model including all main and interaction effects.

Variable	DF	SS	MS	F	Pr (>F)
Rec. Dist.	2	43.33	21.667	24.574	2.58 × 10^−8^
Speed	2	8.26	4.128	4.682	0.0133
Angle	2	15.15	7.574	8.590	0.0006
Rec. Dist: Speed	4	29.24	7.311	8.292	2.74 × 10^−5^
Rec. Dist: Angle	4	15.63	3.908	4.433	0.0036
Speed: Angle	4	16.56	4.140	4.696	0.0025
Rec. Dist: Speed: Angle	8	34.03	4.254	4.824	0.0002
Residuals	54	47.61	0.882		
